# Obituary

**Published:** 1880-07

**Authors:** 


### OBITUARY.

### DIED JUNE 24, M. ROGERS, M. D., D. D. S.

It is with feelings of sadness we announce the death of Melancthon Rogers, M. D., D. D. S., first professor of dental pathology and therapeutics in the Ohio College of Dental Surgery.

Dr. Rogers was in his 84th year, vigorous, and until three days before his decease, in his usual health, when he was suddenly, on Monday night, 21st inst., taken with a severe attack of cholera morbus—he died on the night of the 24th.

Dr. Rogers for many years was the leading dentist of this city, and as a successful practitioner, held high rank wherever known. Fifty years ago he was one of the few medical practitioners West who turned their attention to the specialty of dental surgery; he always identified dentistry with medicine and practiced it as an important branch of surgery, and hence, when the dental college was proposed, threw his whole influence and efficient aid into the enterprise.

As a teacher he was exact and methodical, and could he have remained in the Institution for a few years, would have made his chair one of great prominence.

Every organization for the advancement of science found the Doctor a sincere and ardent supporter, hence in all our early Associations we find his name on the record. Since his retirement from the profession, some ten years since, the Doctor has been living at his country home adjoining Covington, Ky., where, in a sweet home-life, with an accomplished wife and son, his advancing years glided swiftly away. His life for over 65 years bore testimony to the Christian's faith, "he rests in peace."

